# Love at First Glance but Not After Deep Consideration: The Impact of Sexually Appealing Advertising on Product Preferences

**DOI:** 10.3389/fnins.2020.00465

**Published:** 2020-05-29

**Authors:** Fengpei Hu, Qingyuan Wu, Yiwei Li, Weijie Xu, Lei Zhao, Qingzhou Sun

**Affiliations:** School of Management, Zhejiang University of Technology, Hangzhou, China

**Keywords:** sexually appealing ads, utilitarian product, hedonic product, gazing stage, evaluation stage, EEG, product preference

## Abstract

In advertising studies, the impact of sexually appealing advertisements (hereafter “ads”) on consumers’ product preferences is highly controversial. This paper explores (1) how such ads affect consumers’ product preferences at the gazing stage (initial stage of exposure to the ad) and evaluation stage (final product preference), and (2) which type of product (utilitarian vs. hedonic) is more suited to such ads. We used an electroencephalogram to record participants’ product preferences at the gazing stage and self-reported product preferences at the evaluation stage. The results indicated that participants preferred ads with high sex appeal at the gazing stage and ads with low sex appeal at the evaluation stage. Further, compared to utilitarian products, hedonic products were more suited to sexually appealing ads. The findings suggest that the effect of such ads on consumers’ product preferences varies depending on their cognitive stage and the type of product advertised.

## Introduction

Shopping is a necessary part of daily life, and advertising is a common way to advocate products. Instantly capturing consumers’ attention is key to achieving high sales figures. The use of sex appeal in advertising is a common marketing strategy to attract consumers and promote awareness of the product.

### The Controversy of Sexually Appealing Advertisements

Sexually appealing advertisements (hereafter “ads”) promote products using erotic images or sexual words. For example, the pun of “old driver” has two meanings in China. One denotes a driver who has been driving for many years, the other denotes a person who often tells sexual stories or dirty jokes. Owing to the particularity of “sex,” the use of sex appeal in advertising has been highly controversial. On the one hand, it can attract consumers’ attention to products. On the other hand, because of moral factors, people might reject such ads. Therefore, the effect of sex appeal in advertising has been a focus in the advertising literature, and the findings have been rather inconsistent (e.g., Dudley, 1999; LaTour and Henthorne, 1994; Pope et al., 2004; Bushman, 2005). For instance, Dudley (1999) showed that increased physical exposure in ads (e.g., partial/total nudity) weakened consumers’ willingness to buy the products. Bushman (2005) found that such ads reduced consumers’ memory regarding the product brand. LaTour and Henthorne (1994) revealed that consumers’ purchase intention toward jeans decreased when posters with sexual content were used. Inversely, Pope et al. (2004) compared products with sexually appealing ads to those without and found that participants preferred the former.

### Two Possible Factors to Reconcile the Controversy

Two factors may reconcile this controversy. One is the difference between the initial and final stages of product preference. In prior studies, participants’ product preference was measured in an exposed environment. For example, LaTour and Henthorne (1994) evaluated sexually appealing ads by interviewing participants at a mall. Dudley (1999) asked participants to evaluate sex appeal by projecting advertisement images on classroom screens. When answering questionnaires or being observed by others, participants’ product preferences may be influenced by social norms or their sense of shame. It is likely that their final assessment will differ from their initial preference. That is, people may have preferred ads with sex appeal at first glance but did not do so after deep consideration owing to social values. Many researchers have pointed this out (Neeley and Cronley, 2004; Putrevu, 2008). For instance, Putrevu (2008) inferred that sex appeal was a sensitive topic. People may lower their preference for these ads to cater to mainstream values and obtain others’ approval. This may lead to differences in the final preference assessment versus the initial reaction. Neeley and Cronley (2004) found that because of the impact of social expectation bias, people overestimated behavior akin to socially acceptable mainstream values and underestimated behavior considered to be socially unpopular. Neeley and Cronley (2004) highlighted that social expectations often lead to differences between people’s initial and final preferences. Therefore, it is necessary to explore the different effects of sexually appealing ads on product preferences between the initial and final stages.

The other factor is the type of product (utilitarian vs. hedonic). Utilitarian products have effective, useful, functional, necessary, and practical attributes (e.g., a wrench or spoon). Hedonic products have interesting, exciting, thrilling, and enjoyable attributes (e.g., chocolate or perfume) (Dhar and Wertenbroch, 2000; Voss et al., 2003). People often obtain utilitarian or hedonic information from ads. Therefore, appropriate ads enable people to obtain product information, thereby promoting their purchase intention. Many researchers have studied matching of product types with different advertising strategies to achieve better effects (Drolet et al., 2007; Geuens et al., 2011; Bart et al., 2014). For instance, Bart et al. (2014) analyzed data from 59 mobile advertising companies and found that the effect of mobile display ads for utilitarian products was better than for hedonic products. Drolet et al. (2007) found that utilitarian products with rational ads were more popular, while hedonic products with emotional ads were more popular. Geuens et al. (2011) found that emotional ads were more effective in advertising hedonic products. However, they did not have much of an impact on advertising utilitarian products. Ads with sex as the theme can also be considered emotional ads, as they evoke “sexual” emotions to attract consumers. Thus, hedonic products may be more suited to sexually appealing ads.

This paper investigates (1) how sexually appealing ads influence consumers’ product preferences in the initial stage of being exposed to the ads and the final evaluation stage and (2) which type of product (utilitarian vs. hedonic) is more suited to such ads.

### Electroencephalography for Measuring Advertising Effectiveness

In this paper, we used electroencephalography (EEG) to measure initial preference for sexually appealing ads. EEG is an electrophysiological monitoring method that records electrical activity in the brain. It analyzes the changes in people’s brains through event-related potentials (ERP) or spectral content. It can record changes in the brain after a stimulus is presented in real time. Its high temporal resolution can detect people’s brain responses to stimuli in the early, middle, and later stages. The EEG data were used to infer initial preferences for sexually appealing ads. EEG has many applications in advertising research (Ohme et al., 2010). For instance, Ohme et al. (2010) found significant differences in EEGs even though participants consciously reported no difference in preferences. Studies showed that the prefrontal cortex is associated with an individual’s preferences (Ravaja et al., 2013; Telpaz et al., 2015), and prefrontal cortical data can be used to predict subjective preferences and choices. In a study by Ravaja et al. (2013), participants were asked to indicate their purchase intention toward 14 different products, and the results were compared with corresponding EEG data. The results showed that frontal alpha asymmetry may predict consumers’ purchase decisions; a higher perceived need for a product and higher perceived product quality were associated with greater relative left frontal activation. Telpaz et al. (2015) also compared EEG data with subsequent subjective preferences and found that the N200 component of the central electrode of the prefrontal cortex predicted subjective preferences: the more the person preferred the product images, the more positive N200. However, late positive potential and positive slow waves were relatively better than N200. Additionally, Goto et al. (2017) found that N200, an early ERP component, reflected unconditional and automatic process-driven preferences through virtual shopping tasks. Based on previous studies, this paper selected the central electrode fz of the prefrontal lobe as the measuring electrode and the N200 component as the objective index of the initial advertising preference. N200 is a negative-going wave that peaks at 200–350 ms in the post-stimulus time frame and is found primarily over anterior scalp sites. N200 is considered to be related to initial cognitive assessment.

This paper also used the late frontal slow wave (LFSW), which is related to processing cognitive conflict. Studies have shown significant differences in the LFSW between conflict and non-conflict conditions when people process self-preference and social preference (Liu et al., 2004; McCleery et al., 2011). For example, McCleery et al. (2011) observed that the LFSW components were more positive in conflicting situations (e.g., conflicts between self-values and social values) than in non-conflicting situations. We assumed that when preferences for sex appeal conflicted with social norms, the LFSW would be more positive. Therefore, we selected the LFSW component to investigate whether people came into conflict with social expectations while watching sexually appealing ads. Electrodes FP1 and FP2 on the left and right hemispheres were selected as the LFSW electrodes and 400–700 ms as the LFSW time window. Further, self-reported subjective product preference was also recorded as an important final preference.

In addition to analyzing ERP components in EEGs, we also analyzed the brain network. Based on the spatiotemporal organization theory of brain activity (Varela et al., 2001), higher-order cognitive processes and goal-oriented behavior synchronize dynamically with different regions of the brain through neural oscillations of different frequencies to form a unified functional network (Müller and Anokhin, 2012). We constructed a functional brain network based on the phase lag index (PLI) to explore the differences in brain networks triggered by different levels of sex appeal in ads and different types of products. “Small-worldness” has been shown to be one of the basic attributes of brain networks. A high “small-worldness” property indicates higher local clustering and shorter path length in brain networks (Micheloyannis et al., 2006; Zhao et al., 2018). For example, Micheloyannis et al. (2006), by constructing a functional brain network, found that participants with low education had a higher “small-worldness” property than those with higher education, supporting the hypothesis of neural efficiency. They assumed that subjects with low education needed a more optimized network organization structure to achieve cognitive tasks of equal levels of difficulty. Zhao et al. (2018) found through their intertemporal decision-making study that people’s “small-worldness” attribute under the condition of loss aversion was larger than under the condition of benefit, implying that people have greater brain responses under the condition of loss. This means that the higher the “small-worldness” property, the more complex the functional brain network and the greater the brain response. Therefore, we chose “small-worldness” as the evaluation index of the brain network.

### Experimental Overview

In this paper, we used EEG and behavioral reports to record participants’ brain and behavioral responses (e.g., N200 in the initial stage, LFSW and subjective product preference in the final stage) to products with sexually appealing ads. We also examined which type of products (utilitarian vs. hedonic) were more suited to such ads.

## Materials and Methods

### Participants and Design

A total of 25 healthy volunteers participated in this experiment. Data from four subjects were excluded because of errors in the recording of the EEG data. As a result, a total of 21 volunteers were fully analyzed (6 males, 15 females; mean age = 21.19 years ± 2.60). All participants were right-handed with no visual problems and no history of neurological diseases or mental disorders. All participants provided written informed consent prior to the experiment. The study protocol was approved by the Local Ethics Committee of Zhejiang University of Technology. This experiment was a two (sex appeal: high vs. low) × two (product type: utilitarian vs. hedonic) within-participants design.

High and low sex appeal pictures were selected from the study by Black and Morton (2017). Nudity is a common way to present sex appeal in ads. Based on the degree of nudity, we divided advertising pictures into two categories: high and low sex appeal ads.

In this experiment, 34 product images were selected for evaluation. The participants were provided with information on the difference between hedonic and utilitarian products in the following way: *Hedonic products focus on feelings and experiences and are fun, exciting, and enjoyable. Utilitarian products focus on functionality and utility and are effective, beneficial, functional, and practical. Utilitarian products highlight their functional and practical utilities*. Afterward, the participants were asked to evaluate the product type on a seven-point scale (1 = the most utilitarian to 7 = the most hedonic). Thirty volunteers were selected to classify these products. The results were as follows: utilitarian products included vacuum cups, condoms, chewable vitamin tablets, skincare products, shower gels, shampoos, and laundry detergent (*M* = 2.43), while hedonic products included massage oil, cakes, plush toys, potato chips, carbonated drinks, perfumes, music records, and entertainment magazines [*M* = 5.57, *t*(29) = −15.099, *p* < 0.001]. The experimental materials are shown in Table 1. Additionally, we screened the degree of sexual appeal of ads after combining sexually appealing advertising with product types through pre-experiments. Since some products showed little difference between high- and low-appeal ad scores and some products’ high-appeal ad scores were not high, excluding these ads caused differences in the number of ad pictures under different conditions (high sex appeal utilitarian: 6; high sex appeal hedonic: 8; low sex appeal utilitarian: 5; low sex appeal hedonic: 9).

**TABLE 1:** Examples of experimental materials.

Type	Example	Example	Example	Example
Utilitarian products	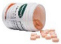 Chewable tablets	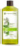 Shampoo	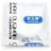 Condoms	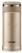 Vacuum cup

Hedonic products	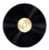 Music album	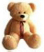 Plush toys	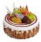 Cake	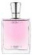 Perfume

### Procedure

Based on the experimental tasks of Telpaz et al. (2015), our experiment was divided into two stages. In the first stage, participants were asked to view each ad image and think about their preferences for the ad, and the corresponding EEG data of the participants were recorded at this stage. The aim was to measure the individual neural activity for each specific ad. In the second stage, the participants were asked to subjectively evaluate their preference for the ads presented in the previous EEG stages.

### Stage 1: EEG Measurement

The experiment was conducted in a soundproof room where the participants sat in front of a computer screen and put on an EEG electrode cap. They were asked to minimize their movements during the EEG experiment. The display of the stimuli and the acquisition of behavioral data were conducted using E-Prime software. All ad images were randomly presented on a standard computer screen. Only one ad image was presented in each trial. The participants were instructed to think about their preference for the ad. During the experiment, they were not required to make any substantive choices or other behavioral responses.

**FIGURE 1:**
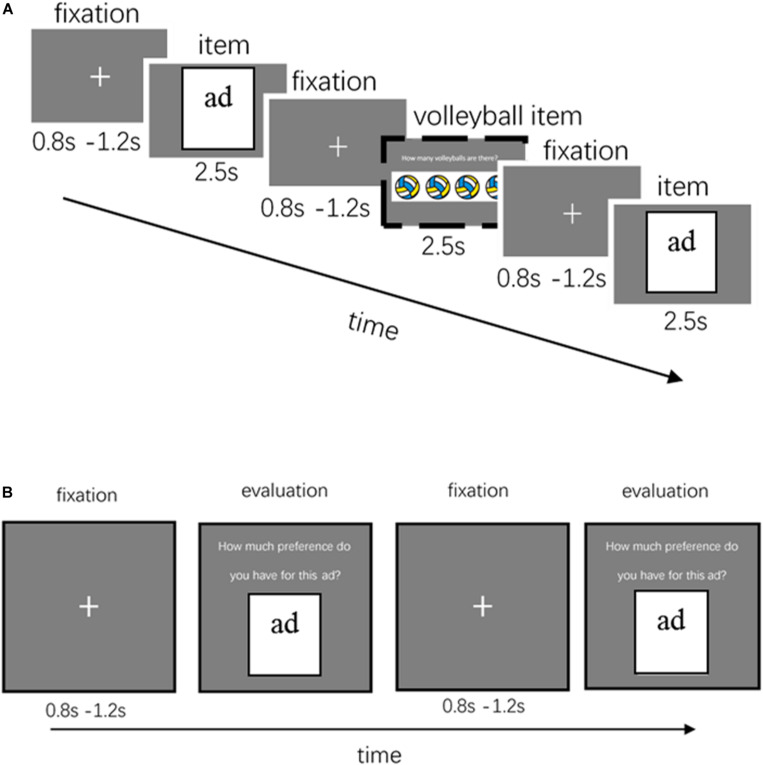
Experimental design. **(A)** EEG Task. After 0.8–1.2 s fixation, the experiment randomly presented an ad; the participants were instructed to think about their preference for the ad. After 2.5 s, the next trial began. After presenting 28 ads, they were asked to answer how many volleyballs were on the screen, as shown in the dashed-border box, and they continued on to the EEG experiment after answering correctly. **(B)** Behavior Task. After 0.8–1.2 s fixation, the experiment randomly presented ads; the participants needed to evaluate their preference for the ad [seven-point scale ranging from “dislike a lot (= 1)” to “like a lot (= 7)”].

Figure 1A depicts the visual presentation of an ad. For each trial, a fixation cross of 800–1,200 ms was first presented, followed by an ad for 2.5 s. The participants were instructed to think about their preference for the ad. To maintain their concentration on the experiment, in 21 random trials (1 of the 21 presentations for each of the 28 items), after the fixation, they were asked to answer how many volleyballs were on the screen. To improve the signal-to-noise ratio, every ad in each block was randomly presented three times. Thus, a block had a total of 84 trials (i.e., high sex appeal utilitarian: 18 trials; high sex appeal hedonic: 24 trials; low sex appeal utilitarian: 15 trials; low sex appeal hedonic: 27 trials; with each ad shown three times). A total of seven blocks was repeated for a total of 588 trials. Participants were allowed a short break between each block (at the end of each block, the screen said that the subject could continue the task whenever he/she was ready by pressing the mouse button). The total duration of the EEG recording stage was 40 min.

For example, after a second of fixation, a high sex appeal hedonic ad was presented for 2.5 s. The participant was instructed to think how much he/she preferred the ad shown. After 28 trials, a picture of volleyballs was presented for 2.5 s. The participant was then asked to answer how many volleyballs were on the screen. At the end of the block, he/she could continue the task when ready by pressing the mouse button.

### Stage 2: Behavioral Stage

At the end of the EEG measurement stage, the EEG electrode cap was removed, and the participant commenced the behavioral task after 10 min of rest. Figure 1B depicts the visual presentation for this task. For each trial, a fixation cross was first presented for 800–1,200 ms, then the screen randomly presented an ad image and the subjects were asked to rate how much they preferred this ad on a seven-point scale ranging from “dislike very much (= 1)” to “like very much (= 7)” for a total of 28 trials (high sex appeal utilitarian: 6 trials; high sex appeal hedonic: 8 trials; low sex appeal utilitarian: 5 trials; low sex appeal hedonic: 9 trials; each ad was present once). Thereafter, the participants filled in their demographic information, such as gender and age, were thanked for their participation, debriefed, and paid.

### EEG Recording and Analysis

The EEG was taken from 64 scalp locations by electrodes mounted on an elastic cap with online reference to the left mastoid. The horizontal electrooculogram was recorded by two electrodes placed laterally on either side of the left and right eyes. The vertical electrooculogram was recorded by two electrodes located above and below the right eye. All electrode impedances were kept below 5 KΩ. All signals were sampled at 500 Hz, and the band-pass filtered frequency range was 0.01–100 Hz.

During the offline analysis, all EEG data were re-referenced to the mean of the left and right mastoids. A 20–Hz (24 dB/oct) low-pass filter was applied to all EEG data. The ocular artifacts in the data were removed via a regression procedure using Neuroscan software. Trials containing EEG sweeps with amplitudes exceeding ±80 μV were excluded. EEG data with a continuous duration of 1000 ms were extracted from each stimulus datum, including a 200 ms pre-stimulus period used as baseline.

In this experiment, the ERP components were analyzed using repeated-measures analysis of variance (ANOVA) in which sex appeal (high sex appeal vs. low sex appeal) and product type (hedonic vs. utilitarian) were used as within-participant factors. The time window of the frontal N200 component selected was 170–270 ms after stimulus presentation as peak amplitude, and the electrode selected was the fz electrode. The selection of the electrode and component time window was based on previous articles and visual inspection of the ERP grand average waveforms.

#### Construction of a Brain Function Network

The standard PLI quantifies the phase synchronization of two different time series by detecting non-zero phase difference coupling. The weighted phase lag index (wPLI) extends the PLI by increasing the phase difference between two time series (Vinck et al., 2011; Hardmeier et al., 2014). Therefore, in this study, we used the wPLI to measure the functional connection between electrodes (nodes). The steps for analyzing the weighted phase lag of each EEG signal were as follows: (1) classifying the EEG signals according to different frequency bands; (2) calculating the EEG signals with the wPLI for each frequency band, each condition, and each subject using HERMES software; (3) determining an appropriate threshold to maintain a connection density of 30% of the connected network in each frequency band (Bullmore and Bassett, 2011); (4) setting the values of connection values in each connection matrix that were greater than the threshold to one and values less than the threshold to zero.

#### Calculating the “Small-Worldness” Property

Based on the brain network connection matrix, we used the network’s small-world index as an analysis index to explore the differences under each condition. The small-world attribute is equal to the clustering coefficient/characteristic path length of the network. Therefore, the clustering coefficient and shortest path length are the two most important parameters used to describe and characterize the small-world topology of a network.

The clustering coefficient reflects the local integration capability of a network. The clustering coefficient indicates the possibility that the neighbors of a certain node are neighbors to each other. The value of the clustering coefficient C*_*i*_* of node *i* is equal to the ratio of the number of edges (*e*_*i*_) connected between the neighbors of the node and the maximum possible number of connected edges [*k*_*i*_ (*k*_*i*_−1)/2].

$$C_{i}=\frac{2e_{i}}{k_{i}\left(k_{i}-1\right)}$$

In this paper, we used the average clustering coefficient of all nodes as the clustering coefficient of the network (Onnela et al., 2005). The shortest path length describes the information transmission capacity within a network. A path with the fewest edges between two nodes, *i* and *j*, is called the shortest path between the two points. The number of edges that the path passes is the shortest path length between nodes *i* and *j*, *l*_*ij*_, and *V* is the set of nodes. The network shortest path length, *L*, describes the average of the shortest path length between any two nodes in the network.

$$L=\frac{1}{N\left(N-1\right)}\sum_{i,j\in v,i\neq j}l_{ij}$$

In this experiment, the method of calculating the “small-worldness” property was based on Hong et al. (2016). We chose the 300–800 ms post-stimulus period for the analysis. Then, we calculated the clustering coefficient and characteristic path length. The “small-worldness” index was defined as the ratio of the clustering coefficient and the characteristic path length.

All data were analyzed using SPSS 21.0, and the significance level was set at 0.05. The Bonferroni method was used to correct the *post hoc* test of multiple comparisons. The important interactions were analyzed with simple-effects models.

## Results

### EEG Results

#### N200

**FIGURE 2:**
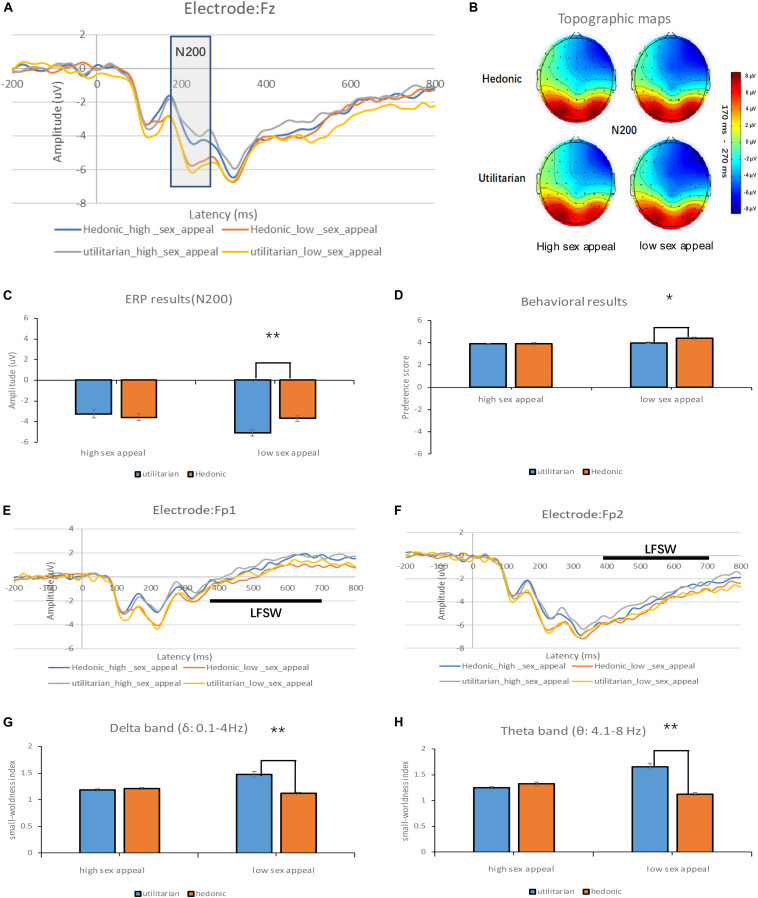
Experimental results. **(A)** The results of the N200 component under four different conditions. There is a significant difference in the N200 peak between 170 and 270 ms after the stimulus was presented. **(B)** Topographic maps under four different conditions. **(C)** The average peak value of the N200 component under four different conditions. **(D)** The subjective preference score under four different conditions. **(E)** The results of the LFSW component under four different conditions. **(F)** The results of the LFSW component under four different conditions. **(G)** The small-worldness index in the delta band. **(H)** The small-worldness index in the theta band. (*p < 0.05, **p < 0.01).

Inspired by Telpaz et al. (2015), we selected the N200 component of the fz electrode for analysis. Telpaz et al. found that the larger the magnitude of N200 in the mid-frontal electrode, the lower the preference for the item tested. The N200 component was analyzed via repeated-measures ANOVA, with sex appeal (high sex appeal vs. low sex appeal) and product type (hedonic vs. utilitarian) used as the within-participant factors. Figure 2A shows the ERP waveforms of the N200 component under the four conditions, and Figure 2B shows the topographic maps of the N200 component under those conditions. The results revealed that the main effect of product type was significant: *F*(1, 20) = 5.315, *p* = 0.032, $\eta_{p}^{2}$ = 0.210. The amplitude was significantly more negative with utilitarian product ads as opposed to hedonic product ads. This indicated that the participants preferred hedonic product ads over utilitarian ones. Further, the results also showed that the main effect of sex appeal was significant: *F*(1, 20) = 9.678, *p* = 0.006, $\eta_{p}^{2}$ = 0.326. The amplitude was significantly more negative for low sex appeal ads (vs. high sex appeal ads). This indicated that participants preferred high sex appeal ads over low sex appeal ads.

Moreover, we also found an interaction between the level of sex appeal and product type: *F*(1, 20) = 14.871, *p* = 0.001, $\eta_{p}^{2}$ = 0.426. As shown in Figure 2C, with low sex appeal, the amplitude of utilitarian product ads was significantly more negative than in hedonic product ads: *F*(1, 20) = 19.70, *p* < 0.001. However, no difference was observed in the amplitudes between utilitarian and hedonic product ads when sex appeal was high: *F*(1, 20) = 1.14, *p* = 0.298. This indicated that when sex appeal was low, the participants preferred hedonic product ads over utilitarian ones, and when sex appeal was high, there was no difference in their preferences.

#### LFSW

Since the LFSW is a late prefrontal slow wave, the FP1 and FP2 electrodes of the prefrontal lobe were selected as the left and right frontal lobe electrodes for analysis based on previous studies (e.g., Sabbagh and Taylor, 2000). We chose the LFSW component to explain whether people exhibit psychological activities that conflict with their own thoughts because of other reasons when they watch sexually appealing ads. The LFSW component was analyzed using repeated-measures ANOVA in which sex appeal (high vs. low sex appeal) and product type (hedonic vs. utilitarian) were used as the within-participant factors. Figure 2E shows the LFSW under the four conditions of the left hemisphere in this experiment. The results revealed that the main effect of sex appeal was significant in the left hemisphere: *F*(1, 20) = 4.731, *p* = 0.042, $\eta_{p}^{2}$ = 0.191. The amplitude was significantly more positive with high sex appeal ads as opposed to low sex appeal ads. However, we did not find any main effect of product type in the left hemisphere: *F*(1, 20) = 0.936, *p* = 0.345, $\eta_{p}^{2}$ = 0.045. There was an interaction between the level of sex appeal and the product type in the left hemisphere: *F*(1, 20) = 0.005, *p* = 0.947, $\eta_{p}^{2}$ = 0.000. Figure 2F shows the LFSW under the four conditions of the right hemisphere in this experiment. The main effect of sex appeal was significant in the right hemisphere: *F*(1, 20) = 6.302, *p* = 0.021, $\eta_{p}^{2}$ = 0.240. The amplitude was significantly more positive with high sex appeal ads than with low sex appeal ads. This indicated that high sex appeal ads may induce larger cognitive conflicts. However, neither a main effect of product type in the right hemisphere nor an interaction between the level of sex appeal and the product type in the right hemisphere were found: *F*(1, 20) = 0.524, *p* = 0.477, $\eta_{p}^{2}$ = 0.026; *F*(1, 20) = 0.251, *p* = 0.622, $\eta_{p}^{2}$ = 0.012.

#### “Small-Worldness” Property

We selected the “small-worldness” property developed by Hong et al. (2016) as the basic indicator of the brain network. The higher the “small-worldness” property, the more complex the functional brain network and the more brain areas that need to be activated. The “small-worldness” property was analyzed using repeated-measures ANOVA in which sex appeal (high sex appeal vs. low sex appeal) and product type (hedonic vs. utilitarian) were used as the within-participants factors. Figure 2G shows the “small-worldness” property of the delta band under the four conditions of this experiment, while Figure 2H shows the “small-worldness” property of the theta band. The findings were as follows:

(1) Delta band (δ: 0.1–4 Hz): The results revealed that the main effect for the product type was significant [*F*(1, 20) = 33.993, *p* = 0.001, $\eta_{p}^{2}$ = 0.630]. The “small-worldness” property was significantly higher in utilitarian product ads than in hedonic ones. There was no significant difference in sex appeal [*F*(1, 20) = 2.450, *p* = 0.133, $\eta_{p}^{2}$ = 0.109]. We also found an interaction between sex appeal and product type [*F*(1, 20) = 6.863, *p* = 0.016, $\eta_{p}^{2}$ = 0.255]. When the ad’s sex appeal was low, the “small-worldness” property was significantly higher in utilitarian product ads than in hedonic ones [*F*(1, 20) = 14.88, *p* = 0.001]. However, there was no significant difference when the ad’s sex appeal was high [*F*(1, 20) = 0.2, *p* = 0.658].
(2) Theta band (θ: 4.1–8 Hz): The results revealed that the main effect for the product type was significant [*F*(1, 20) = 22.900, *p* = 0.000, $\eta_{p}^{2}$ = 0.534]. The “small-worldness” property was significantly higher in utilitarian product ads than in hedonic ones. There was no significant difference in sex appeal [*F*(1, 20) = 2.941, *p* = 0.102, $\eta_{p}^{2}$ = 0.128]. We also found an interaction between sex appeal and product type [*F*(1, 20) = 15.511, *p* = 0.001, $\eta_{p}^{2}$ = 0.437]. When the ad’s sex appeal was low, the “small-worldness” property was significantly higher in utilitarian product ads than in hedonic ones [*F*(1, 20) = 25.91, *p* < 0.001]. However, there was no significant difference when the ad’s sex appeal was high [*F*(1, 20) = 0.98, *p* = 0.333].
(3) Alpha band (α: 8.1–12 Hz): The results revealed that there was no significant main effect for product type [*F*(1, 20) = 4.146, *p* = 0.055, $\eta_{p}^{2}$ = 0.172] or sex appeal [*F*(1, 20) = 0.142, *p* = 0.710, $\eta_{p}^{2}$ = 0.007]. There was no interaction between sex appeal and product type [*F*(1, 20) = 0.361, *p* = 0.554, $\eta_{p}^{2}$ = 0.018].
(4) Beta band (β: 12.1–30 Hz): The results revealed that the main effect for the product type was significant [*F*(1, 20) = 5.980, *p* = 0.024, $\eta_{p}^{2}$ = 0.230]. The “small-worldness” property was significantly higher in utilitarian product ads than in hedonic ones, and the main effect for sex appeal was significant [*F*(1, 20) = 10.281, *p* = 0.004, $\eta_{p}^{2}$ = 0.340]. The “small-worldness” property was significantly higher when the ad’s sex appeal was low than when it was high. There was no interaction between sex appeal and product type [*F*(1, 20) = 0.361, *p* = 0.554, $\eta_{p}^{2}$ = 0.079].

#### Behavioral Results

A two (sex appeal) × two (product type) repeated measurement ANOVA on ad preference was conducted. The results revealed that the main effect of sex appeal was significant *F*(1, 20) = 5.644, *p* = 0.028, η_*p*_^2^ = 0.220], indicating that the participants likely preferred low sex appeal ads (*M* = 4.20, *SD* = 0.76) over high sex appeal ads (*M* = 3.91, *SD* = 0.79). The main effect of the product type was not significant [*F*(1, 20) = 2.690, *p* = 0.117, $\eta_{p}^{2}$ = 0.119].

The interaction between sex appeal and product type was marginally significant [*F*(1, 20) = 3.901, *p* = 0.062, $\eta_{p}^{2}$ = 0.163]. As shown in Figure 2D, when the ad’s sex appeal was low, participants preferred hedonic product ads over utilitarian ones [*F*(1, 20) = 5.62, *p* = 0.033]. However, there was no difference in preference between the utilitarian and hedonic product ads when their sex appeal was high [*F*(1, 20) = 0.06, *p* = 0.815]. This was consistent with the EEG results.

## General Discussion

This paper explored the effects of sexually appealing ads on utilitarian and hedonic product preferences. The results showed that the participants preferred high sex appeal ads at the gazing stage but preferred low sex appeal ads at the evaluation stage. Further, compared to utilitarian products, hedonic products were more suited to sexually appealing ads.

### Sex Appeal and Its Effect on Advertising

Our paper contributes to the research on the controversy of sexually appealing ads. According to the behavioral results, people preferred low sex appeal ads over high sex appeal ones at the final evaluation stage, which is consistent with some previous studies (LaTour and Henthorne, 1994; Dudley, 1999). According to the ERP results, people preferred high sex appeal ads over low sex appeal ones at the initial gazing stage. The results of the LFSW indicated that sexually appealing ads induced larger cognitive conflicts.

We divided the process of experimenting with the ads into three steps: watching, thinking, and evaluating. Participants watched the ads first, then thought about the relevant factors of their preference, and finally made a preference evaluation. The N200 component, which was used to predict people’s preferences, is an event-related-potential component. N200 is a negative-going wave that peaks at the 200–350 ms post-stimulus time frame. We used the N200 component as a preference assessment for the watching stage. The subjective assessment results belonged to the evaluation stage. The LFSW component is a late component that is generated by a person’s independent thinking. Therefore, it can be considered as an indicator of mental activity in the thinking stage. In this paper, there was a difference in the LFSW between ads with different appeal intensity. We inferred that when people evaluated high sex appeal ads, conflicts arose because of their initial preferences and social expectations. However, the sexual content in the ads with low sex appeal was still within the socially acceptable range, which caused people to have different LFSWs.

Therefore, the psychological changes at the thinking stage caused the difference in preferences between the watching and evaluation stages. Further, sex appeal differences were observed in the beta band brain network. Low sex appeal ads showed stronger task-related modulations of the beta band brain networks than high sex appeal ads. This means that the beta band is related to preference evaluation and that, compared to high sex appeal ads, people may need more brain regions activated to participate in the evaluation of low sex appeal ads. This implies that people have a more in-depth understanding of low sex appeal ads. Sexually appealing ads may attract consumers at first, but excessive sexual content makes them lose interest in the ad; consumers do not make a deep evaluation of the ads. Thus, our participants had poor behavior evaluation scores and a low “small-worldness” property. In general, using sex appeal in advertising can immediately attract people’s attention; as the degree of sex appeal increases, the attraction gets stronger, as verified by the ERP results. However, consumers may have negative reactions to excessive sexual content or may wish to set an example for others and thus do not further entertain high sex appeal ads. This is also evidenced by the “small-worldness” property of the brain network. As a result, the final evaluation deviated from the initial preference. This observation has also been verified by previous studies (Neeley and Cronley, 2004).

Cultural differences between the East and West may be another important reason for the inconsistent preferences in the early and later stages of reaction to strongly appealing ads. Many previous studies explored the impact of the cultural differences between East and West (e.g., Oyserman and Lee, 2008). Western culture is considered individualistic, encouraging people to express their inner state or feelings. Therefore, when people in such countries browse through sexually appealing ads, they may be more willing to follow their heartfelt thoughts and directly express their own internal state or feelings. Eastern cultures, however, highlight conservatism, observe social norms, and stress social harmony (Oyserman and Lee, 2008). Asian cultures tend to be collectivist, encouraging people to adhere to social values (Tsai et al., 2007). Therefore, when viewing such ads, people in Eastern countries initially have a higher preference for sexually appealing ads but later change their initial thinking to meet social expectations.

### Product Type and Sex Appeal

Our findings contribute to the literature on the effects of advertising for different types of products. Based on the ERP and behavioral results, low sex appeal ads were more effective in advertising hedonic products but not in advertising utilitarian ones. Consistent with emotional ads, sexually appealing ads were more suited to advertising hedonic products (Drolet et al., 2007; Geuens et al., 2011). Additionally, utilitarian and hedonic product ads were evaluated poorly when their sex appeal was high. This is consistent with previous studies: excessive sex appeal reduces people’s purchase intention (LaTour and Henthorne, 1994; Dudley, 1999).

### Theoretical and Practical Implications

Theoretically, the current paper explored two possible factors regarding the controversy about sexually appealing ads in previous studies. Our results suggested that the effect of sexually appealing ads on consumers’ product preferences varies depending on their cognitive stage and the type of product advertised. People preferred ads with high sex appeal at the gazing stage but ads with low sex appeal at the evaluation stage. Further, compared to utilitarian products, hedonic products were more suited to sexually appealing ads.

Practically, using sex appeal in advertising is one of the commonly used strategies for marketers. Our findings suggest that for ads with high sex appeal, people may prefer them at the initial stage but not at the final stage because of social pressure. For ads with low sex appeal, their combination with hedonic products may produce positive advertising effects. During offline shopping where consumers are exposed to other individuals, high sex appeal ads may not be a smart way to advertise products as opposed to in online shopping, where the environment is relatively private. Additionally, advertising hedonic products with low sex appeal ads may be more effective than employing such ads for utilitarian products.

### Limitations and Future Directions

#### Social Expectation Bias

Social expectation bias led to the reversal of stated preferences to sexually appealing ads. In public, the participants stated that they preferred the low sex appeal ads. Future studies should employ different process measures (e.g., “think aloud” protocols or post-rating interview data) to justify this explanation. There should be a high correlation between social desirability and the stated preference for low sex appeal ads. Open-ended responses may reveal participants’ dislike for high sex appeal advertising for moral reasons. Another method would be to use procedures such as guaranteed private and anonymous data collection to minimize social expectation bias and emotional response display rules (e.g., Ekman, 2006). We believe that the stated preference reversal may be weaker or disappear altogether if privacy and anonymity are guaranteed^1^.

#### Sample Size

We determined our sample size (*N* = 21) based on previous studies that used EEG (Barry et al., 2003; Daurignac et al., 2006) and studies related to ours (Telpaz et al., 2015), however, our small sample size is still a limitation. We call for future studies to test our findings using a larger sample.

#### N200

We used the N200 component as an early indicator of initial preference. Although prior studies suggest that it predicts initial subjective preferences, recent studies have shown that N200 is a weak measure of preference (Goto et al., 2017). Future studies can further test the effectiveness of N200 as an indicator of preferences or use other components to measure early preferences.

## Data Availability Statement

The datasets presented in this article are not readily available because the data cannot be shared for privacy restrictions. Requests to access the datasets should be directed to QW, 850703661@qq.com.

## Ethics Statement

The studies involving human participants were reviewed and approved by Local Ethics Committee of Zhejiang University of Technology. The patients/participants provided their written informed consent to participate in this study.

## Author Contributions

FH, LZ, and QS conceived and designed the experiments. YL and QW performed the experiments. YL, QW, and WX analyzed the data. FH and QS drafted the manuscript. QS and QW revised the manuscript. QS edited the manuscript.

## Conflict of Interest

The authors declare that the research was conducted in the absence of any commercial or financial relationships that could be construed as a potential conflict of interest.

## Footnotes

1 We thank the reviewer who brought this limitation to our attention.
